# Templated 3ʹ terminal fluorescent labeling of RNA using Klenow DNA polymerase

**DOI:** 10.1016/j.mex.2024.102925

**Published:** 2024-08-28

**Authors:** Mary N. Mwangi, Nathan J. Baird

**Affiliations:** Saint Josephʹs University, Philadelphia, PA, USA

**Keywords:** Fluorescent labeling of RNA, 3ʹ end labeling of RNA, Klenow DNA polymerase, Templated 3ʹ terminal fluorescent labeling of RNA using Klenow DNA polymerase

## Abstract

A long-standing challenge in the study of RNA structure-function dynamics using fluorescence-based methods has been the precise attachment of fluorophores to structured RNA molecules. Despite significant advancements in the field, existing techniques have limitations, especially for 3ʹ end labeling of long, structured RNAs. In response to this challenge, we developed a chemo-enzymatic method that uses Klenow DNA polymerase to label RNAs. In this method:

• Klenow DNA polymerase adds an amino-modified nucleotide to the 3ʹ end of the RNA, guided by the DNA oligonucleotide template.
• An NHS-ester dye is then conjugated to the amino-modified RNA, forming a covalent amide bond.
• For highly structured RNAs, DNA oligonucleotides complementary to the RNA disrupt pre-existing intramolecular RNA structures.

This methodological advancement enables site-specific incorporation of a single modified nucleotide at the 3′ terminus of various RNA substrates, irrespective of their length or secondary structure. The user-friendly nature of the technique, with minimal modifications required for different RNA targets, makes it readily adaptable by a broad range of researchers. This approach has the potential to significantly improve the development of functionalized RNA for various applications.

## Graphical abstract

**Figure:**
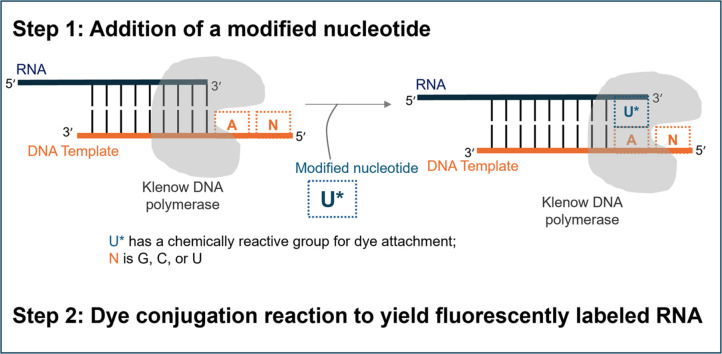

Specifications table

**Table:** 

Subject area:	Biochemistry, Genetics and Molecular Biology
More specific subject area:	RNA Labeling
Name of your method:	Templated 3ʹ terminal fluorescent labeling of RNA using Klenow DNA polymerase
Name and reference of original method:	Not applicable
Resource availability	See **Method details** description

## Background

The low intrinsic fluorescence of RNA presents a fundamental challenge for fluorescence-based visualization. To address this limitation, fluorophores are attached site-specifically to RNA. However, the attachment of fluorophores to specific locations on an RNA molecule can be hindered by the secondary and tertiary structures of the RNA. Furthermore, ensuring that fluorophore attachment does not perturb these structures is essential, as structural alterations may compromise RNA functional activity and interactions.

Currently available labeling methods are best suited for short RNA fragments and are not readily amenable to labeling longer, structured RNA molecules. While some methods have been developed to label long RNAs [[1], [2], [3]], many other current techniques have significant limitations. For example, chemical methods of labeling nucleic acids through solid-phase synthesis or periodate oxidation are more applicable to the labeling of shorter fragments of largely unstructured RNA, less than 100 nucleotides, with a limited number of modified building blocks [[4], [5], [6], [7]]. Enzymatic-based methods offer an alternative approach to label long and structured RNAs. However, enzymatic-based methods have limitations, including the tendency to incorporate multiple untemplated fluorophores [[8], [9], [10]]. These considerations underscore the ongoing need for refining and expanding techniques in RNA labeling.

We have developed and optimized a method that utilizes Klenow DNA polymerase in a templated manner to attach a single fluorophore to the 3ʹ end of RNAs. Our method is designed to be user friendly, inexpensive, and straight-forward. We show that our method is applicable to incorporating a single fluorescent label to the 3ʹ end of short, long, and structured RNAs that have been chemically synthesized or *in vitro* transcribed.

## Method details

### Nucleic acid design and synthesis

Five RNAs were selected as our model RNAs to demonstrate the effectiveness of our 3ʹ terminal end labeling method (Table 1 and Fig. S1). These 5 RNAs were chosen due to their diverse characteristics in length, structure, and synthesis method (Table 1 and Fig. S1).

**Table 1:** RNAs and DNAs used in this study. The two-nucleotide 5′ overhang in the DNA templates is highlighted in blue. The templating nucleotide, complementary to the modified nucleotide, is underlined.

ᶧ Nucleic acids were chemically synthesized (Syn), purchased from either IDT DNA Technologies or Dharmacon.

ᶴ Nucleic acids were prepared by *in vitro* transcription (IVT) using T7 RNA polymerase.

Four of these RNAs comprise a portion of the 3ʹ triple helix of the lncRNA MALAT1 construct M1^AB^, as previously reported [11]. These RNAs are also described in detail in our research article (Mwangi et al., manuscript in press, ACS Biochemistry). The fifth RNA to be labeled consists of a segment of the HIV RRE RNA [12,13]. Herein, we describe our method development using three chemically synthesized RNAs and then demonstrate the application of our method to two *in vitro* transcribed RNAs.

We designed and purchased three chemically synthesized RNAs of variable lengths designated as M1^A^ (22 nt), M1^A_Extended^ (26 nt), and M1^A_Shortened^ (16 nt) to use as substrates (Table 1). Our fourth and fifth RNAs, M1^B^ RNA (54 nt) and HIV RRE (233 nt), were *in vitro* transcribed using T7 RNA polymerase and subsequently used as substrates in our method (Table 1 and Table 2). Briefly, *in vitro* transcribed RNA constructs were transcribed from amplified PCR products using T7 RNA polymerase as described [14]. The RNAs were purified using a 6% preparative denaturing polyacrylamide gel. Bands having the RNA of interest were excised and subjected to electroelution at 200 volts for 4 hours (Elutrap, GE). The RNA was then precipitated in 3X v/v of absolute ethanol and 0.1X v/v of 3M sodium acetate, pH 5.3, at -20 °C overnight. The RNA was pelleted by centrifugation at 16,000 * g for 1 hour, subsequently resuspended in DEPC-treated water, and stored at -20 °C until further use.

**Table 2:** HIV RRE RNA sequence and complementary DNA templates and DNA oligonucleotide sequences. The HIV RRE RNA region complementary to the DNA template is indicated by a double underline. Consistent with Table 1, the two-nucleotide 5′ overhang in the DNA templates is highlighted in blue, and the templating nucleotide complementary to the modified nucleotide is underlined.

ᶴRNA (bold sequence) was prepared by *in vitro* transcription (IVT) using T7 RNA polymerase.

ᶧDNA template and DNA capture oligonucleotide were chemically synthesized (Syn), purchased from IDT DNA Technologies.

We designed and purchased chemically synthesized complementary DNA templates for the five RNAs (Table 1 and Table 2), which upon hybridization with the RNA substrate contain a two-nucleotide overhang on the 5ʹ end of the template oligonucleotide [15]. This two-nucleotide overhang contained adenine as the first templating base (Table 1 and Table 2, blue underlined nucleotide) and cytosine as the last base. Our choice of a two-nucleotide overhang was motivated by previous research by Huang and Szostak [15]. Their work demonstrated that a two-nucleotide overhang led to a 20-fold greater incorporation of a radiolabeled nucleotide compared to having a single nucleotide overhang. [15]. We also demonstrate this higher incorporation yield using a 2-nucleotide overhang as compared to a 1-nucleotide overhang in Fig. S3. For the 3 M1^A^ RNAs, we designed DNA templates to hybridize the entire RNA sequence. However, we designed and tested four DNA templates for the 54-nucleotide M1^B^ RNA, with variable lengths spanning 10, 15, 22, and 35 nucleotides (Table 1). The 22 and 35 nucleotide templates yielded sufficient hybridization to achieve the highest yield for the n+1 reaction. For the 233-nucleotide HIV RRE RNA, we designed a 47-nucleotide DNA template (Table 2). In addition to the DNA template, we also designed a 27-nucleotide DNA capture oligonucleotide for the long HIV RRE RNA (233 nucleotides) (Table 2). This capture oligonucleotide was designed to disrupt pre-existing structure near the 3ʹ terminus to enable efficient hybridization of the template oligonucleotide.

### Choosing a modified nucleotide

**Fig. 1:**
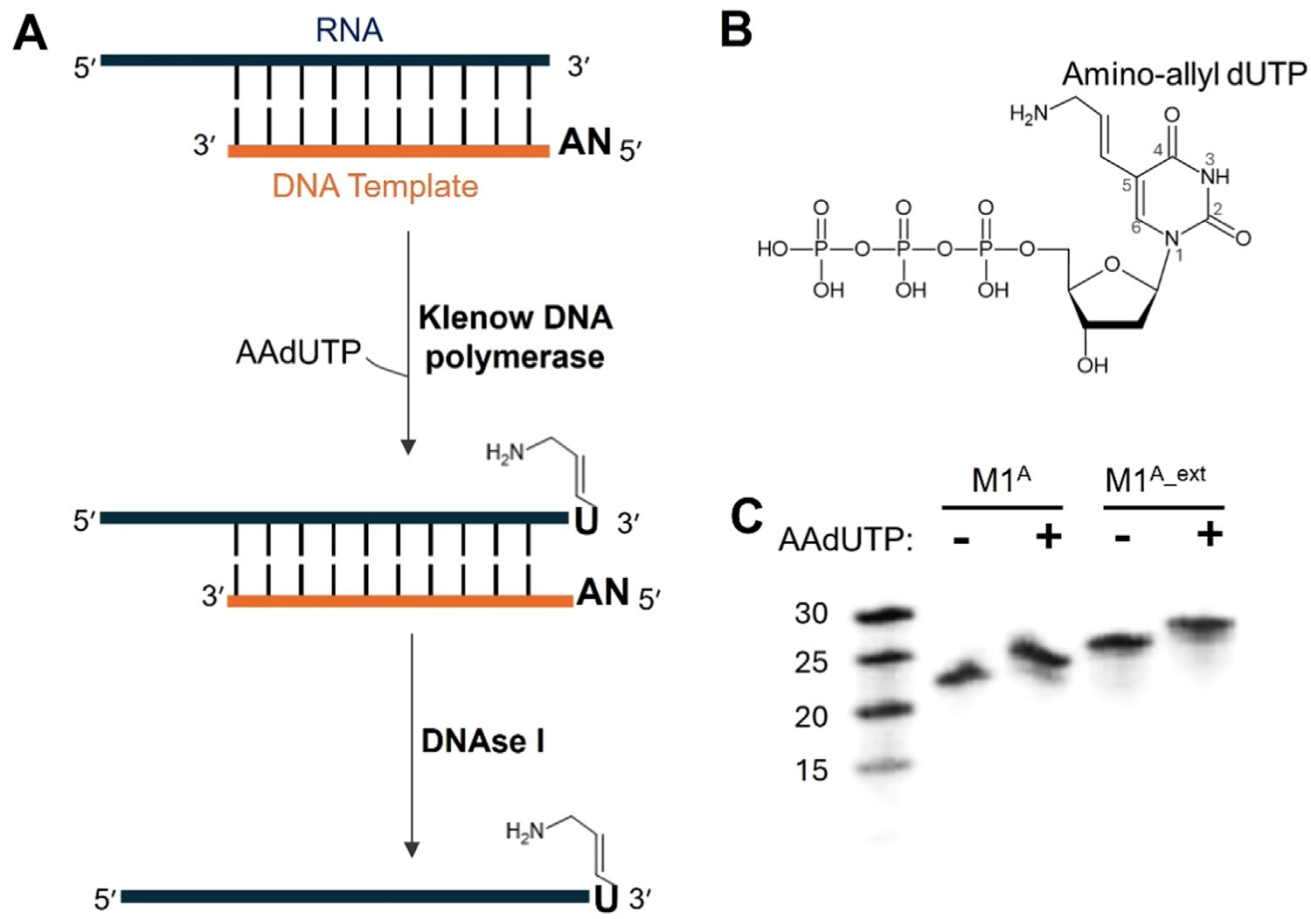
**Templated addition of amino-allyldUTP (AAdUTP) to the 3ʹ end of RNA using Klenow DNA polymerase.** A. Schematic illustration of the n+1 addition reaction and subsequent DNase I treatment to obtain 3ʹ modified RNA. B. The structure of the amino-allyl dUTP nucleotide, including the amino-allyl modification on position 5. C. A 20% denaturing analytical gel stained with Sybr Gold showing the addition of AAdUTP to M1^A^ and M1^A_Extended^ RNAs. The M1^A^ samples appear slightly twisted, which may be due to a minor issue during gel electrophoresis. This potential artifact does not alter the interpretation of the data.

We chose to use a commonly available modified nucleotide, 5-[3-aminoallyl]-2ʹ-deoxyuridine-5ʹ-triphosphate, AAdUTP (ThermoFisher) (Fig. 1A and B). This nucleotide features a deoxyribose sugar that is easily accommodated in the DNA polymerase active site and a modified uridine base that contains a primary amino group extending from position 5 on the base (Fig. 1B). This amino group, as we shall see in the later sections, is important when conjugating to different NHS-ester fluorophores. It has previously been shown that this modified nucleotide can be incorporated into DNA using various DNA polymerases, including Klenow DNA polymerase, for the non-radioactive labeling of DNA [[16], [17], [18]]. In addition to the AAdUTP modified nucleotide, we also explored the addition of different modified nucleotides by Klenow enzyme to the 3ʹ end of the RNA (Fig. S2), thus showcasing the wide applicability of using this method to attach different labels and modifications to RNA. The primary aim of this protocol is to establish a method for the fluorescent labeling of RNA.

**Fig. 2:**
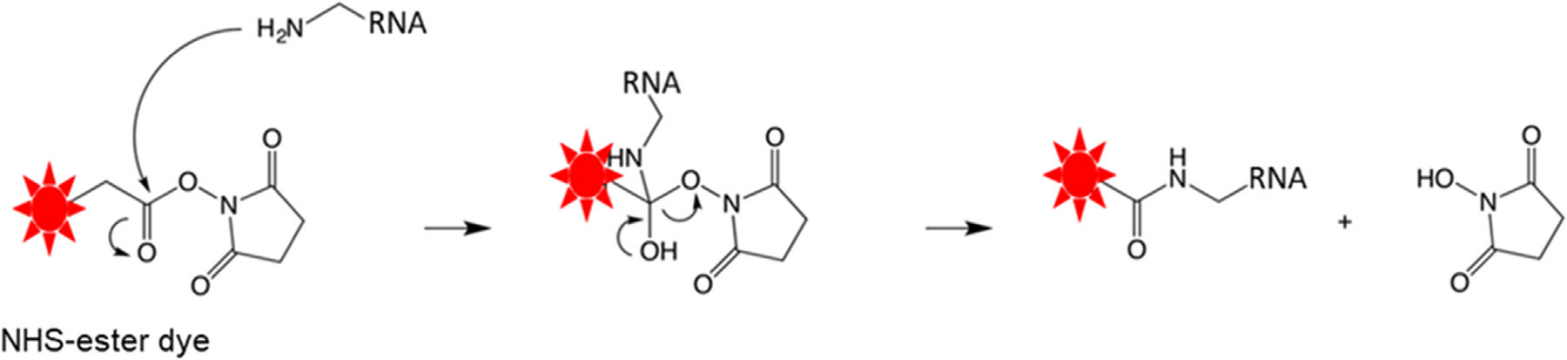
Scheme showing the RNA-NHS ester dye conjugation reaction.

### Protocol

#### Optimizations

The first step in this protocol involves the incorporation of the modified nucleotide AAdUTP to the 3ʹ terminal end of the RNA. We refer to this as the n+1 addition reaction. The AAdUTP is enzymatically incorporated in a templated manner using Klenow DNA polymerase (NEB) (Fig. 1). We performed the following optimization experiments to increase the efficiency of the n+1 addition.

1. We determined the concentration range of the enzyme, DNA template, and AAdUTP that would support the experimental efficiency of yield. (Fig. S3 and Fig. S4).
2. We also ran time course experiments to determine the minimum incubation time needed for reliable and efficient n+1 addition (Fig. S3).
3. Additionally, by varying the length of the DNA templates that hybridize to the RNA of interest, we obtained the best DNA-RNA annealing pair under the reaction conditions (Fig. S3 and Fig. S4).

A summary of the findings from the optimizations is discussed in the Supplementary Information section (see Additional Notes, Fig. S3, and Fig. S4).

*Step 1: N+1 addition reaction*

In our optimized method, RNA, DNA template, AAdUTP, and Klenow were combined in a Klenow buffer containing 50 mM NaCl, 10 mM MgCl_2_, 1 mM DTT, and 10 mM Tris-HCl, pH 7.9, to a final volume of 250 µL (Table 3). The reaction mixture was incubated at 37 °C for a minimum of 2 hours to enable the addition of AAdUTP to the 3ʹ end of the RNA. We recommend an incubation time range of 2-8 hours based on reaction conditions.

**Table 3:** N+1 addition reaction components.

Component	Stock concentration			Final concentration	
RNA		5	µg/µL	0.4	µg/ µL
DNA template		400	µM	Variable*	µM
AAdUTP		50	mM	1	mM
Klenow		5	U/µL	0.02	U/µL
Klenow buffer		10	X	1	X

⁎ *Note*: Concentration of the DNA template used was twice the molar concentration of the RNA.

After the incubation, the n+1 reaction mixture was treated with RNase-free DNAse1 (Promega) to a final concentration of 1U of enzyme per 1 µg of RNA. This reaction requires the addition of CaCl_2_ to a final concentration of 10 mM. This reaction mixture was incubated at 37 °C for 1 hour to facilitate complete digestion of the DNA template (Fig. 1A). This DNase treatment step is added to simplify the purification of RNA_n+1_ for short RNAs.

To purify the RNA_n+1_ from the excess mononucleotides and digested DNA, we loaded the samples on pre-run 6% denaturing polyacrylamide gels that were run at 30 watts for variable time depending on the size of the RNA. The RNAs were identified by UV shadowing. It is expected that only one band will be visible because the DNA template should have been degraded in the previous step. The observed band corresponds to RNA_n+1_. The band containing the RNA was excised from the gel. RNA was eluted from the gel band passively by the “crush and soak” method using a buffer containing 300 mM sodium acetate and 1 mM EDTA and overnight incubation at 4 °C. The RNA in solution was recovered from the gel pieces by filtering using a 0.22-micron filter. Subsequently, the RNA was precipitated using ethanol precipitation. The RNA_n+1_ pellet was recovered by centrifugation and resuspended in DEPC-treated water. The total RNA recovered was estimated by Nanodrop spectrophotometer.

As a quality control check, we ran an analytical denaturing PAGE of the RNA_n+1_ to assess the efficiency of the n+1 addition reaction (Fig. 1C). For this analysis, 0.1 µg of the purified RNA_n+1_ was diluted with water to a volume of 10 µL and mixed with 10 µL of RNA loading dye (formamide, bromophenol blue, and xylene cyanol). The sample was loaded onto a 20% denaturing PAGE, which was run at 30 watts for a variable amount of time depending on the length of the RNA. Gels were stained with Sybr Gold (ThermoFisher) and visualized under UV exposure using a Biorad Imager. We prepared a sample of the unmodified RNA, designated as RNA_n_, to run on the gel as a control. We observed a retardation in the movement of RNA_n+1_ when compared to RNA_n_, corresponding to the addition of a single nucleotide to the RNA (Fig. 1C). The percentage of n+1 incorporation was estimated to be greater than 90% by comparing the signal of the RNA_n+1_ in the sample to the total signal using ImageJ software.

*Step 2: Dye conjugation reaction*

The second step of this method involves the conjugation of NHS-ester cyanine dyes to the 3ʹ end of the RNA. To prepare for the dye conjugation reaction, 1 mg of cyanine dye (Cytiva) was resuspended in DMSO to a final working concentration of 20 mM. This was then divided into 10 µL aliquots, which were stored at -20°C until used.

The purified RNA_n__+1_, containing a 3ʹ-terminal amino-allyl dUTP, was next utilized as a substrate in the dye-conjugation reaction. For this reaction, the resuspended cyanine dye was added in 10-fold molar excess to the RNA. Additionally, 100 mM sodium phosphate buffer, pH 8.4, and 30% v/v DMSO were added to the RNA-dye mixture to a final volume of 100-200 µL (Table 4). The reaction mixture was incubated at room temperature in the dark for at least 4 hours to facilitate the conjugation of the dye to the amino linker group on the RNA. This conjugation reaction proceeds when the NHS ester of the dye is activated by a nucleophilic attack by the amine group of the modified RNA (Fig. 2). The subsequent transition state is stabilized by the polar aprotic DMSO solvent. Additionally, the DMSO solvent destabilizes the hydroxyl group of the transition state, resulting in the formation of a stable amide bond that covalently links the cyanine dye to the aminoallyl-modified RNA_n__+1_ with the release of the NHS group (Fig. 2) [19,20].

**Table 4:** Sample labeling reaction. Reactions were carried out in a final volume of 100-200 µL with approximately 100 µg of RNA n+1. It is important to note that due to our reaction design, the molar concentrations of the RNAs varied.

Component	Final concentration
RNA	1 X
Dye*	10 X
DMSO	30% v/v
Phosphate buffer, pH 8.4	0.1 M

⁎ *Note*: The dye concentration is equivalent to a 10-fold molar excess of the RNA concentration.

Following overnight incubation, the RNA was subjected to ethanol precipitation as described above to eliminate excess unreacted dye and the NHS leaving group, which remain in the supernatant. The RNA was recovered by centrifugation. The RNA pellet was colored owing to the attachment of the dye in the previous steps (red for Cy3, blue for Cy5). The RNA was resuspended in DEPC-treated water. The recovered RNA was subjected to a second round of dye conjugation to increase the labeling efficiency.

As the final purification step, we carried out gel purification to remove unlabeled RNA, aggregated dye, excess dye, and NHS leaving group from the second labeling reaction. In this step, the labeling reaction was mixed with an equal volume of 2X RNA loading buffer and loaded onto a pre-run denaturing PAGE gel. The gels were then run at 30 watts for approximately 2-4 hours, depending on the size of the RNA. The covalently attached cyanine dye present in the RNA served as a visual marker to track the migration through the gel. When the gel run time was completed, the gels were observed under visible and UV light. The labeled RNA was clearly separated from the unlabeled RNA for the short M1^A^ RNAs using this method. This clear separation results from the increase in size of the RNA upon fluorophore conjugation; therefore, the labeled RNA migrates slower than the unlabeled RNA_n+1_. The band corresponding to the labeled RNA was excised and subjected to “crush and soak” as described above. This was then subjected to ethanol precipitation and centrifugation to recover the labeled RNA. The recovered labeled RNA was resuspended in an appropriate volume of DEPC-treated water. Aliquots of 10 µL each were prepared and stored at -20°C.

#### Evaluating the efficiency of the dye-conjugation reaction

**Fig. 3:**
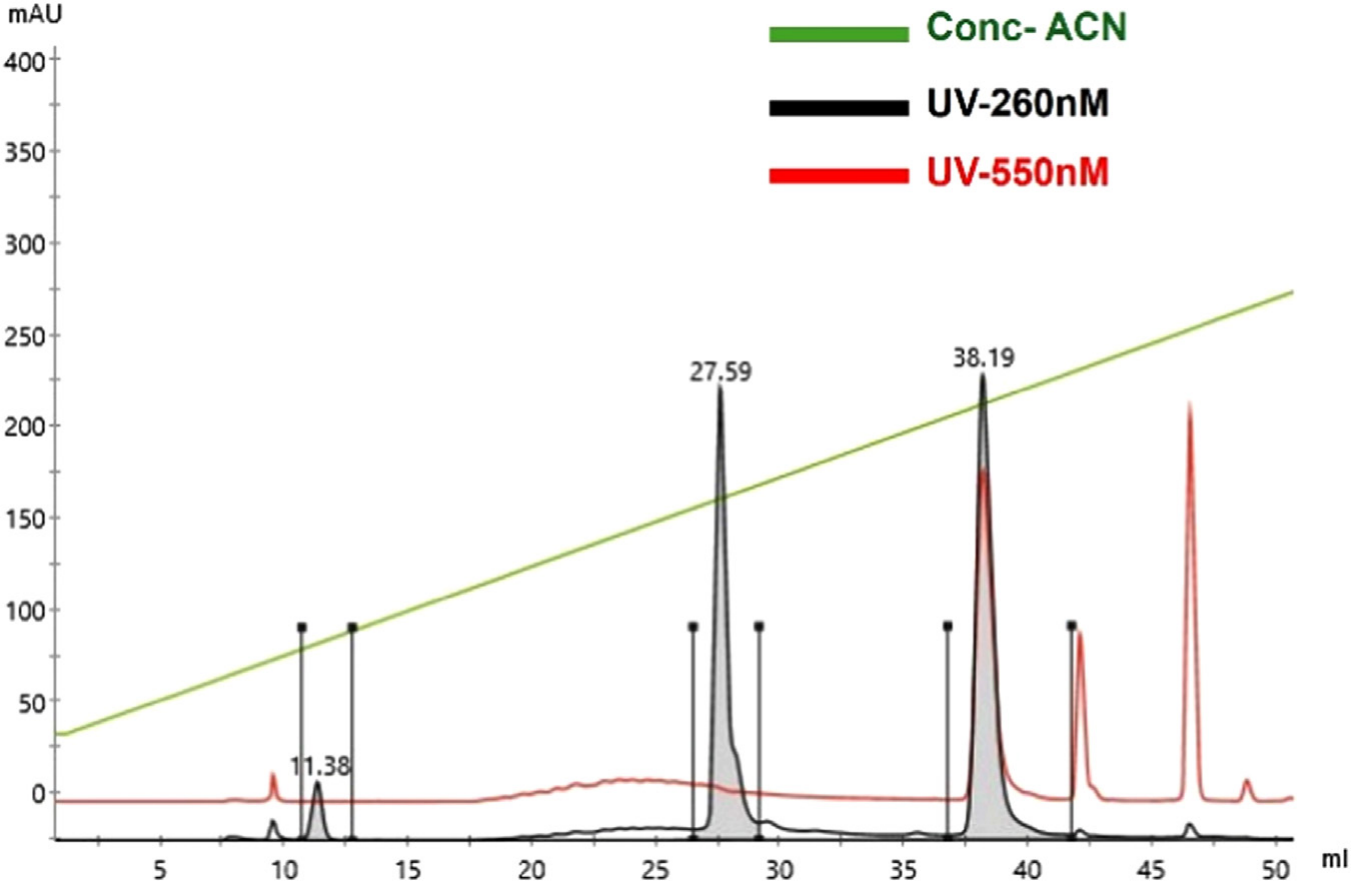
**Chromatographic analysis of labeling efficiency via reverse phase HPLC for the labeling of M1**^**A**^**RNA.** At increasing acetonitrile concentrations (green), the unlabeled RNA eluted first (27.59 ml), as indicated by the 260 nm signal. The labeled RNA eluted at approximately 18% acetonitrile (35.19 ml), as indicated by the coincident 260 nm and 550 nm signals. The labeling efficiency was estimated by calculating the ratio of the peak area at 260 nm of the labeled RNA to the sum of the areas of the labeled and unlabeled peaks at 260 nm.

To assess the labeling efficiency of this method, labeled M1^A^ RNA was loaded onto an analytical C-8 reverse phase HPLC column (XDB-C8 Agilent) equilibrated in 5% v/v acetonitrile in 95% v/v 50 mM triethyl ammonium acetate. A constant flow rate of 2 ml/min and a gradient of 5%-20% acetonitrile was used in this analytical run. Peaks were tracked at 260 nm and 550 nm to follow the RNA and cyanine dye, respectively (Fig. 3). At approximately 11% acetonitrile concentration, a major peak with a signal only at 260 nm eluted. This represents the unlabeled RNA. At higher concentrations of about 18%, labeled RNA exhibiting peaks at both 260 nm and 550 nm eluted. Excess dye eluted at 20% acetonitrile concentration and was characterized by having only a 550 nm peak. The labeling efficiency was estimated by comparing the labeled RNA peak area to the sum of the 260 nm peak areas for both the labeled and unlabeled RNA. The yield was determined to be approximately 50%.

## Method validation

RNA molecules often have complex secondary and tertiary structures. The size and structure of the RNA molecule play a pivotal role in determining the most appropriate labeling strategy. Ensuring that labeling does not perturb these structures is essential, as structural alterations may compromise RNA activity. End-labeling of RNA is a promising strategy for a wide range of applications when the 3ʹ end does not alter the folding or native structure of the RNA. Different methods may be better suited for short RNA fragments compared to longer RNAs with more secondary and tertiary structures. Some labeling methods may not work well with structured RNA or may alter its natural folding.

To validate that our method was applicable to the labeling of structured RNAs, we labeled M1^B^ (54 nucleotides) and HIV RRE (233 nucleotides) RNAs at the 3ʹ end with cyanine dyes. M1^B^ RNA potentially forms a simple secondary structure with the terminal 3ʹ end of the RNA involved in a long duplex stem (Fig. S1). The HIV RRE RNA has a complex RNA structure composed of multiple stems around a central junction. Additionally, a long stem composed of multiple paired regions and loops forms at the terminal ends of this RNA (Fig. S1) [12,13].

We optimized our reaction scheme to label these 2 structured RNAs. For the M1^B^ RNA, we explored the annealing of DNA templates of varying lengths ranging from 12, 17, 24, and 37 nucleotides (Fig. S4). Additionally, we also explored increasing the concentration of the DNA template up to a 10-fold excess over the RNA to facilitate efficient annealing of the template to the RNA. Analysis of gel shift assays of M1^B^ RNA binding to DNA template shows optimal hybridization with template length greater than 17 nucleotides at a concentration 2-fold greater than that of the RNA (Fig. S4).

**Fig. 4:**
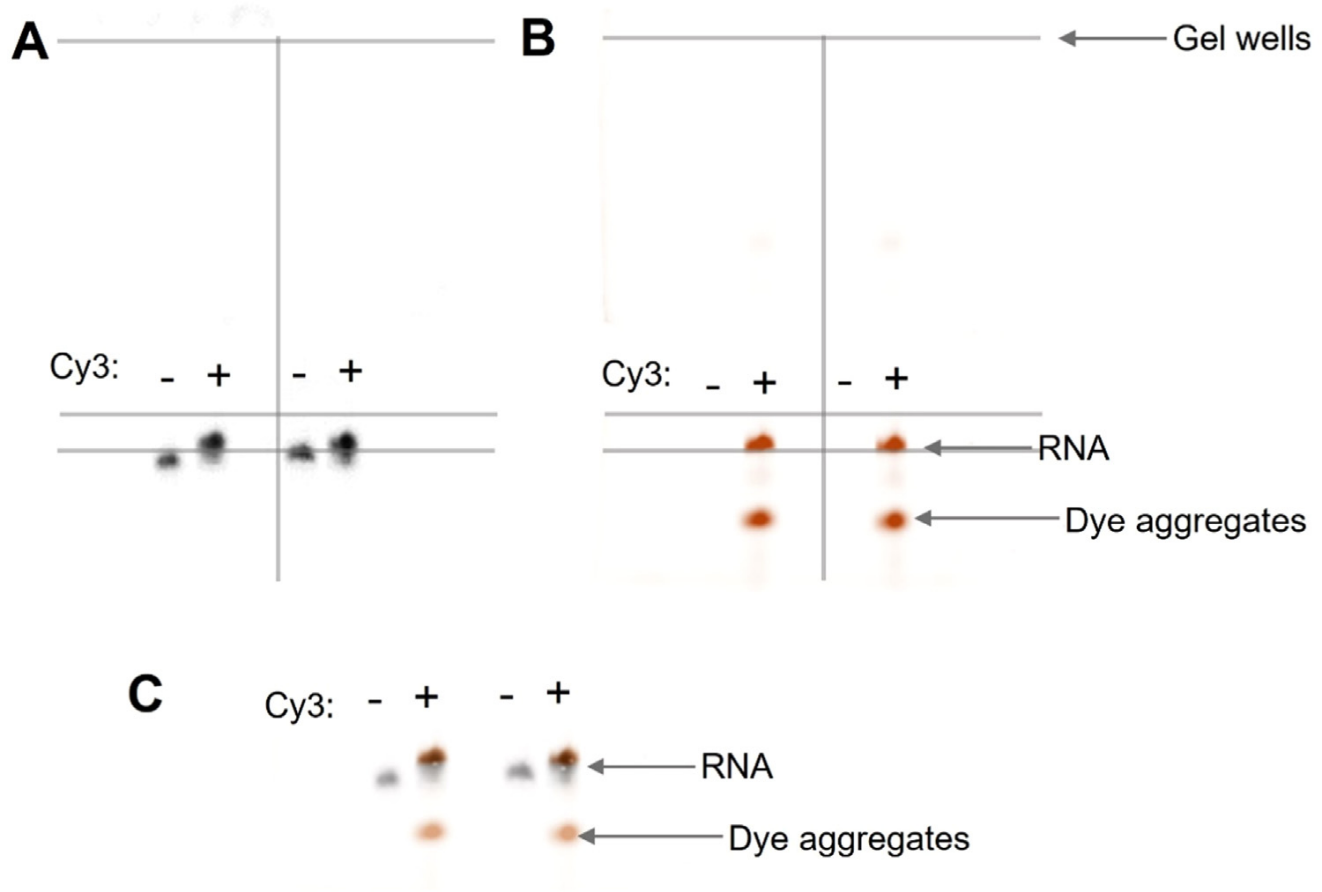
**Gel analysis of the M1**^**B**^_**n+1**_**RNA conjugation to Cy3.** A. A 6% denaturing gel stained with ethidium bromide, showcasing the control M1^B^_n+1_ band and the labeled M1^B_Cy3^. B. The same gel was fluorescence imaged using the Amersham Typhoon Cy3 filter. C. The overlay of the two images shows the labeled RNA and dye aggregate bands.

To facilitate the annealing of the DNA template to the structured M1^B^ RNA, we included a heating step in which, the RNA and DNA template were incubated in Klenow buffer at 65°C for 5 minutes, followed by a slow cooling to room temperature for 30 minutes. Following this annealing process, we added Klenow enzyme and AAdUTP to the reaction and followed the protocol described in the previous section to successfully label M1^B^ RNA with a cyanine dye at its 3ʹ end. We validated the labeling of M1^B^ by running it on a 6% denaturing gel. This gel was stained with ethidium bromide for UV imaging (Biorad Gel Doc) and fluorescently imaged with a Cy3 filter (Amersham Typhoon) (Fig. 4A and Fig. 4B respectively). An overlay of the 2-gel images shows an overlap of the Cy3 signal with the RNA signal (Fig. 4C), suggesting that the RNA was successfully labeled. In addition to the M1^B_Cy3^, we also observed some dye aggregates present in our sample, which can be purified away during gel electrophoresis.

**Fig. 5:**
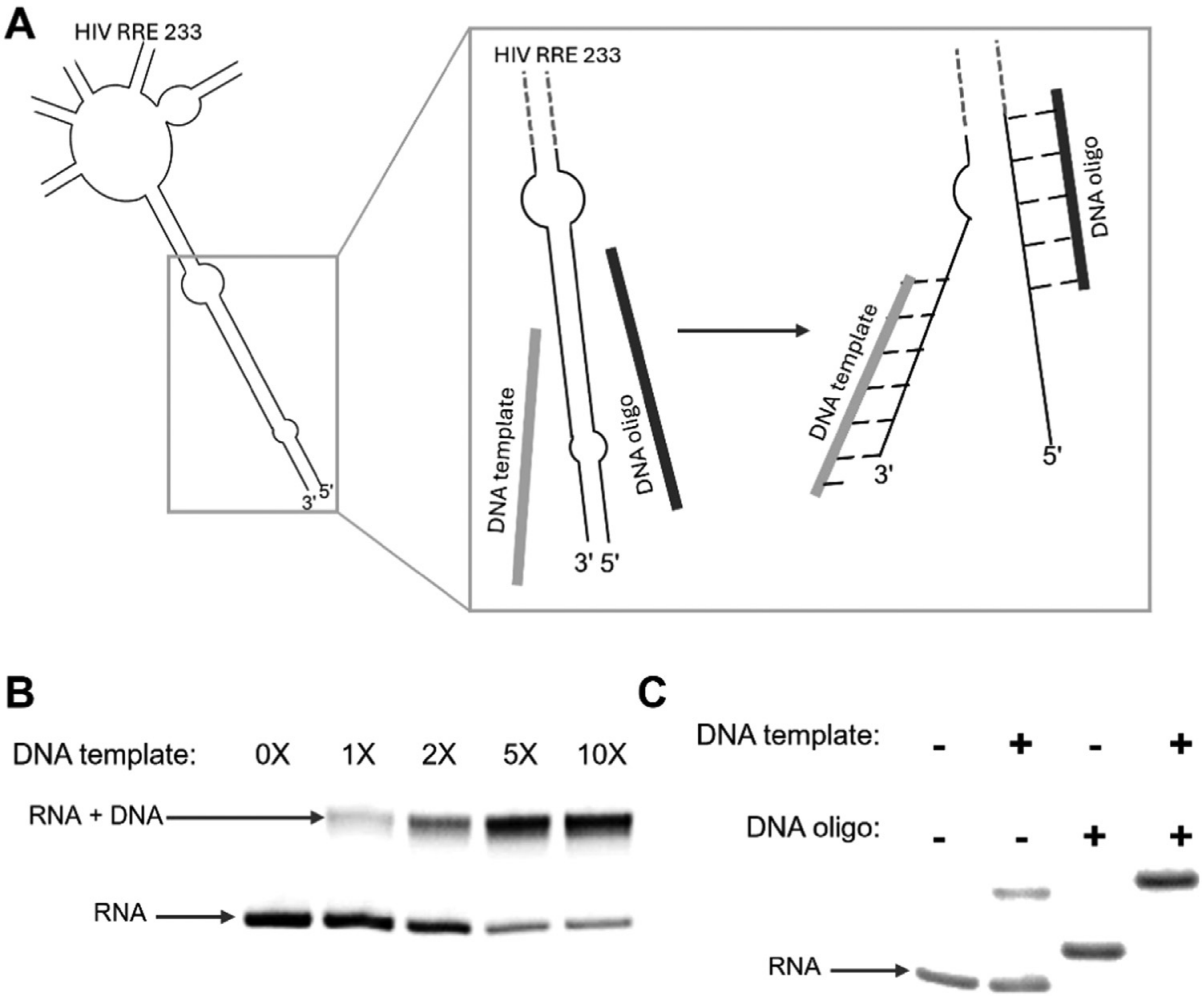
**Labeling structured RNA requires the disruption of pre-existing structures using DNA capture oligonucleotides.** A. Cartoon depiction showing the DNA template and DNA capture oligonucleotide both hybridize to the HIV RRE RNA to disrupt the stem paired region at the terminal end of the RNA. B. A 6% native gel showing the hybridization of increasing concentrations of DNA template to HIV RRE RNA. C. A 6% native gel showing the hybridization of DNA template and DNA capture oligonucleotide to HIV RRE RNA.

Similar to the structured M1^B^ RNA, the annealing of the DNA template to the highly structured HIV RRE RNA required additional steps. Here, we modified the n+1 reaction prior to the heating step as follows; we added a DNA capture oligonucleotide complementary to the structured region near the termini of the RNA. This oligo was designed to capture and disrupt the RNA structure (Fig. 5). The DNA capture oligonucleotide worked in tandem with the DNA template to disrupt the pre-existing RNA structure at the 3ʹ-terminal end, thus facilitating the accessibility of the 3ʹ end of the RNA to the Klenow enzyme for efficient n+1 addition (Fig. 5A). The DNA template alone could not disrupt the pre-existing intramolecular structure in HIV RRE, even at high concentrations of up to 10-fold excess under the heating conditions we tested (Fig. 5B). It was only with the addition of the DNA capture oligonucleotide that the RNA effectively hybridized to the DNA template (Fig. 5C).

**Fig. 6:**
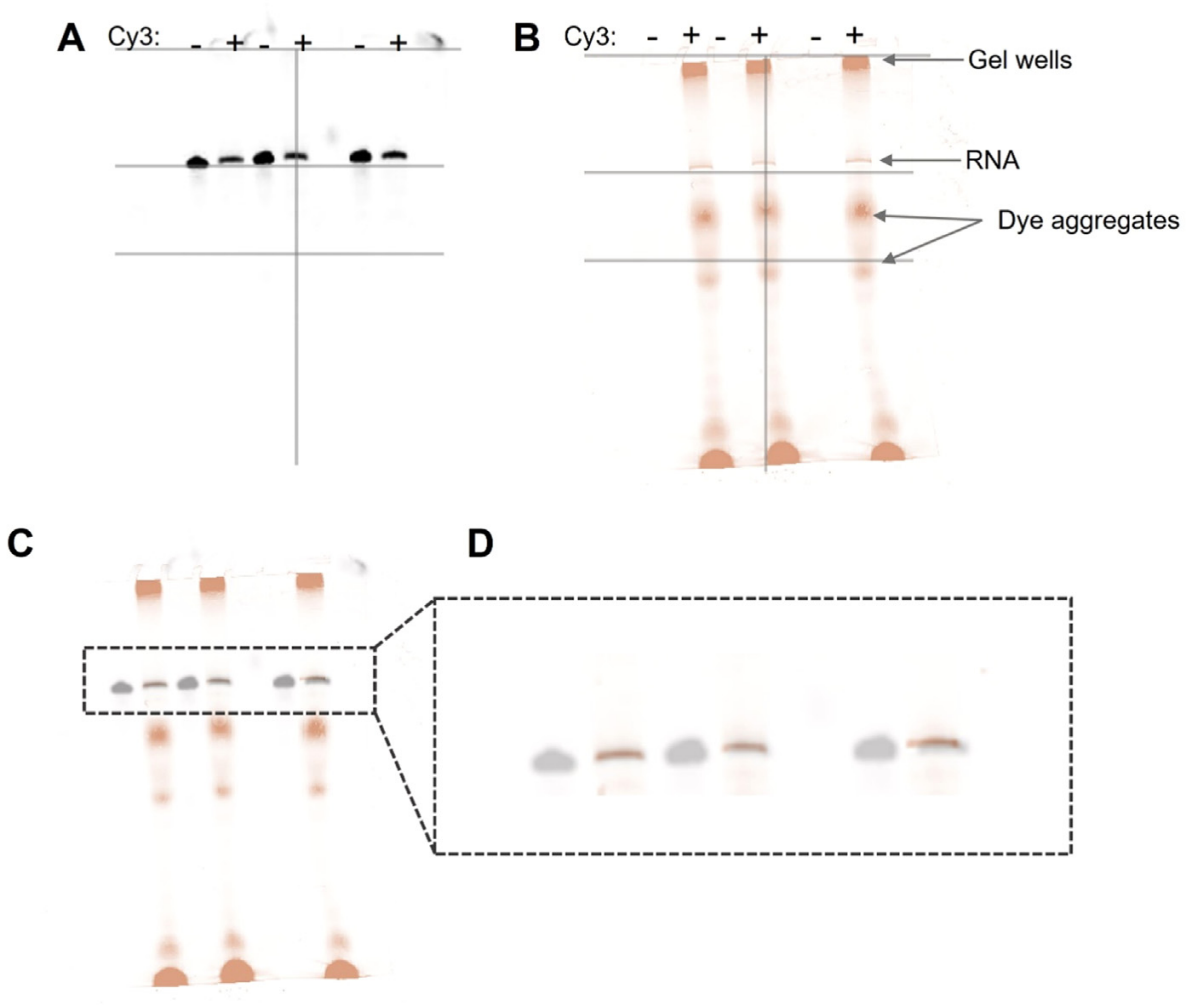
**Gel analysis of the HIV RRE**_**n+1**_**RNA conjugation to Cy3.** A. A 6% denaturing gel stained with ethidium bromide, showcasing control RNA, HIV RRE_n+1_, and the labeled HIV RRE_Cy3_. B. The same gel was fluorescence imaged using a Cy3 filter. C. The overlay of the two images shows the labeled RNA and dye aggregate bands. D. Gel blow up image of the overlay showing the labeled RNA.

Using this combined approach of capture oligo together with the template oligo, the HIV RRE RNA was labeled with Cy3 dye. We validated this labeling using gel analysis similar to the labeling of M1^B^. Our analysis of the gel image stained with ethidium bromide revealed a single band corresponding to the HIV RRE 233 RNA (Fig. 6A). When this same gel was imaged using a fluorescence gel imager, we observed multiple bands (Fig. 6B). By overlaying the 2 gel images, we determined the bands representing the labeled RNA and the aggregated excess dye (Fig. 6C and D).

**Fig. 7:**
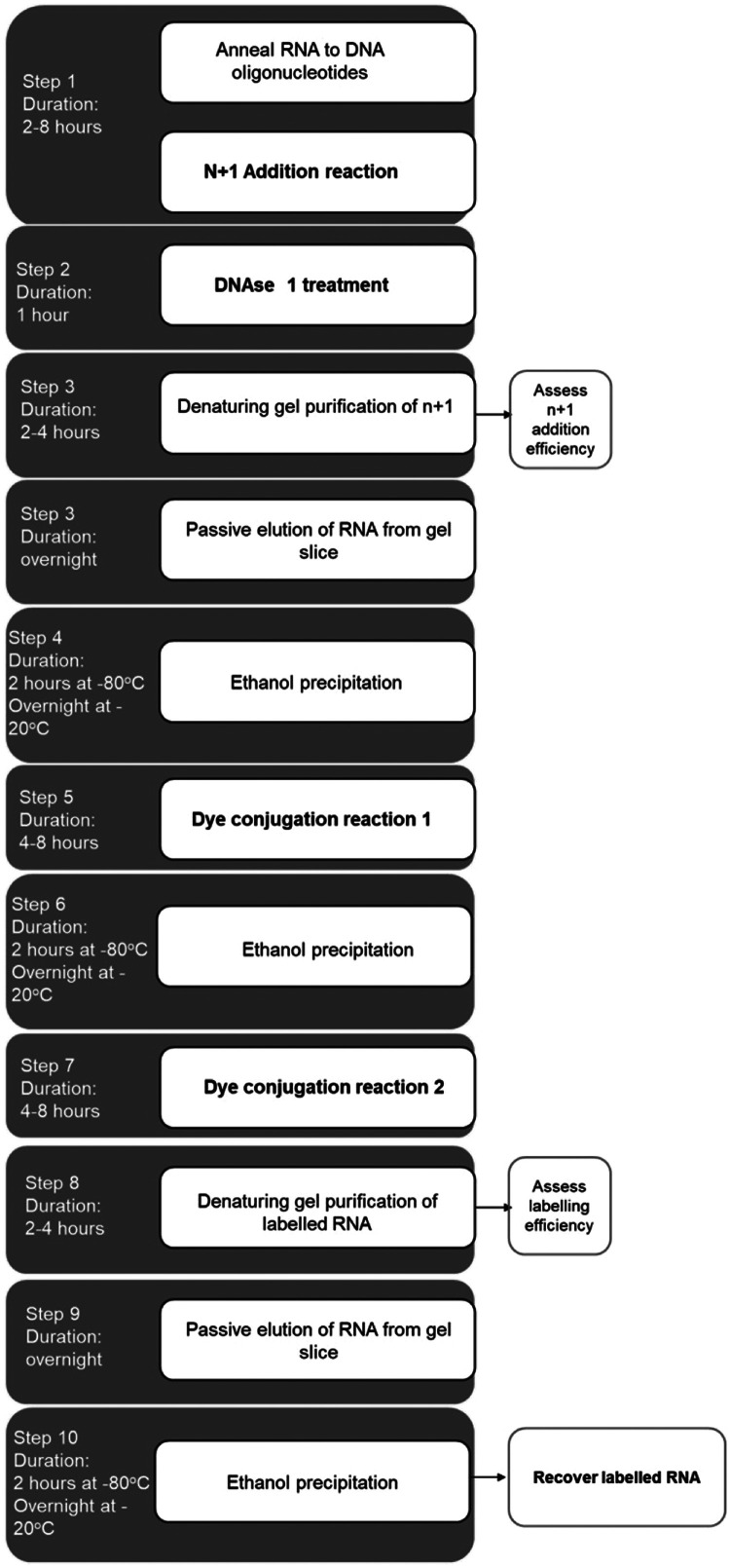
Step-by-step method for the 3ʹ end labeling of RNA using Klenow DNA polymerase.

**Fig. 8:**
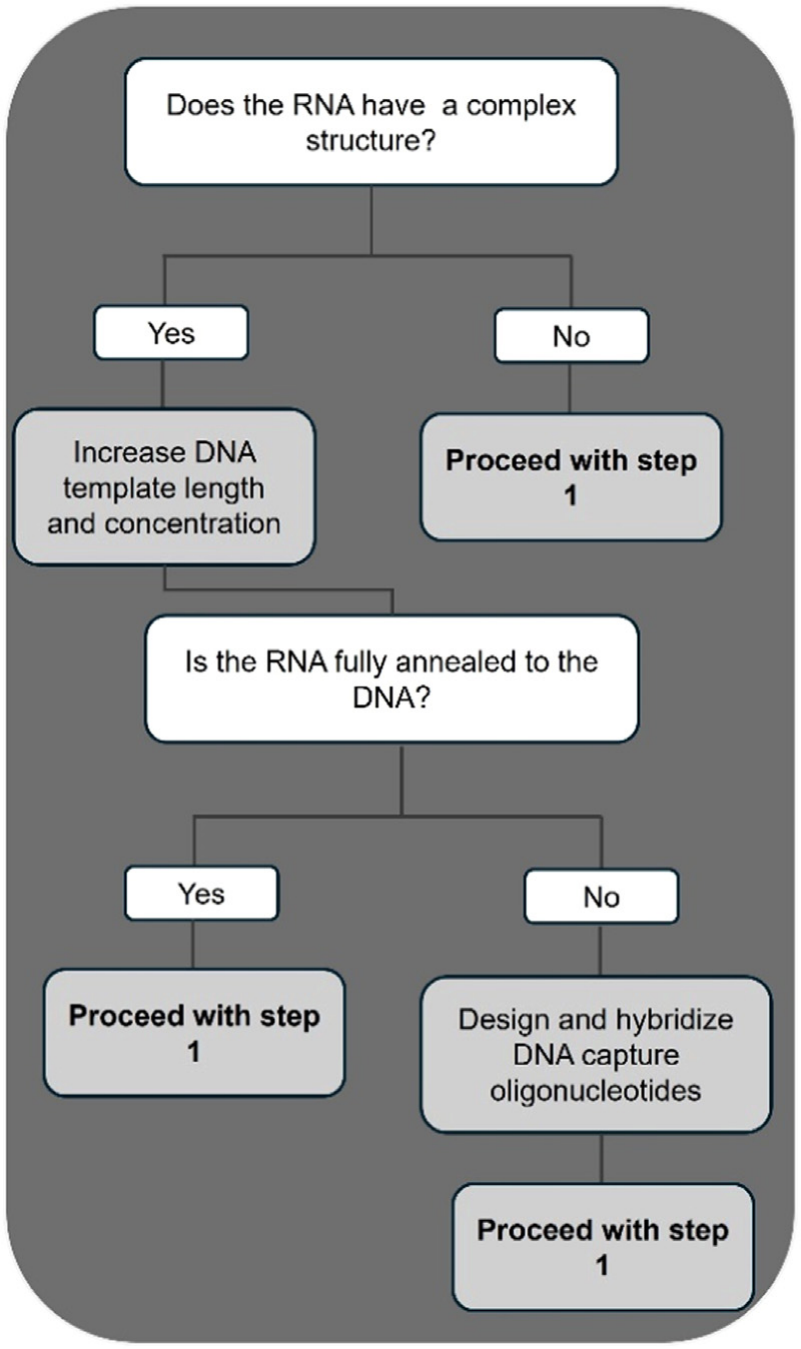
A guide to optimizing the labeling of structured RNA.

In conclusion, we have developed a method for the templated addition of a single label at the 3ʹ terminus of RNA using Klenow DNA polymerase. A summary of the step-by-step details of this method, including the duration of each step, is presented in Fig. 7. Additionally, we provide a resource guide for optimizing the n+1 addition of modified nucleotides to structured RNA (Fig. 8).

### Limitations

Our current method for fluorescently labeling structured and non-structured RNAs at the 3′ terminus is effective, especially given the small amount of material needed for most fluorescence-based experimental applications. For any applications requiring larger amounts of labeled RNA, our method may not be optimal; there are limitations to our approach owing to the use of gel electrophoresis and passive elution in our purification steps. If higher recovery yields are needed, reversed phase chromatography may be an appropriate purification technique.

While NHS-ester chemistry provides a user-friendly method for our small scale labeling protocol, future studies could investigate the incorporation of click chemistry reactive groups as an alternative labeling strategy, which may offer increased versatility and utilization of this method. Click-type reactions, such as strain-promoted alkyne-azide cycloaddition (SPAAC), offer several advantages, including potentially higher labeling yields and faster reaction times. Commercially available azide-labeled (d)UTP analogs may be compatible with Klenow Fragment for incorporation, as suggested by our preliminary experiments showing results of n+1 addition to incorporate analogues of UTP (Fig. S2). Additionally, readily available dyes with complementary functional groups can be employed for SPAAC reactions. Future exploration of click chemistry labeling strategies are worth pursuing for the potential of incorporating diverse fluorophores and streamlining labeling strategies.

## CRediT authorship contribution statement

**Mary N. Mwangi:** Methodology, Validation, Formal analysis, Investigation, Writing – original draft, Writing – review & editing, Visualization. **Nathan J. Baird:** Conceptualization, Resources, Writing – review & editing, Supervision.

## Declaration of competing interest

The authors declare that they have no known competing financial interests or personal relationships that could have appeared to influence the work reported in this paper.

## Data Availability

Data will be made available on request.

## Appendix Supplementary materials

Image, application 1
