# Supplementary material for: Templated 3ʹ terminal fluorescent labeling of RNA using Klenow DNA polymerase
Source: MethodsX. 2024 Aug 28;13:102925. doi: 10.1016/j.mex.2024.102925 (PMC11407071; doi:10.1016/j.mex.2024.102925)
Supplement: Supplementary file 1 [file mmc1.pdf]

Article information

Article title
Templated 3′ terminal fluorescent labeling of RNA using Klenow DNA polymerase

Authors
Mary N. Mwangi and Nathan J. Baird*

Affiliations
Saint Joseph's University, Philadelphia, PA, USA

Corresponding author's email address and Twitter handle
nbaird@sju.edu

Keywords
Fluorescent labeling of RNA; 3′ end labeling of RNA; Klenow DNA polymerase

Related research article
Mary N. Mwangi, Michael J. Yonkunas, Abeer A. Ageeli, Kayleigh R. McGovern-Gooch, Sevde A. Yilmaz, and Nathan J. Baird, *A newly identified peripheral duplex anchors and stabilizes the MALAT1 triplex*, ACS Biochemistry, 2024, https://doi.org/10.1021/acs.biochem.4c00156.

Supplementary material *and/or* additional information

Additional notes: N+1 addition reaction optimizations

1. Klenow concentration was varied from 0.1 U/µL to 0.4 U/µL in a n+1 addition reaction that was incubated for 2 hours at 37°C. At 0.1 U/µl we observed the least amount of AAdUTP incorporation, ~ 95%. Maximal incorporation (100%) was observed when the enzyme concentration was increased to 0.2U/ µL with no further efficiency achieved at higher concentrations of Klenow (0.3 and 0.4 U/ µL) (**Fig. S3B**).
2. AAdUTP concentrations ranging from 0.2 mM to 1 mM were evaluated in an n+1 addition reaction time course experiment (2-, 4-, 6-, 8-, and 16-hour incubation) (**Fig. S3C**).
    a. Following a 2-hour incubation period, we show that maximal addition (~95%) is observed at 1 mM AAdUTP. At lower concentrations of AAdUTP 0.2, 0.4, and 0.6 mM, the incorporation rate was approximately 20%, 50%, and 70% respectively.
    b. The optimal conditions maximizing the addition of AAdUTP to the RNA were seen when the AAdUTP concentration was 1 mM with incubation at 4+ hours (100%). The reaction efficiency did not increase when higher incubation times were evaluated. A similar efficiency was also seen when 0.6 mM AAdUTP and a 16-hour incubation were used.
3. DNA template concentrations ranging from 1-fold to 10-fold of the RNA concentration were evaluated in native gel shift assays. For the shorter RNAs $M1^{A}$, $M1^{A\_Shortened}$, $M1^{A\_extended}$,and $M1^{B}$, a 2-fold excess of the DNA template was sufficient to maximize the hybridization of the template with the RNA of interest (**Fig. S4**). However, for the longer HIV RRE RNA, increasing the DNA template concentration up to 10-fold was not successful in achieving maximal hybridization (**Fig. 5**).
4. We varied the length of the DNA template for $M1^{B}$ and HIV RRE RNA as described in the earlier section on nucleic acid design and synthesis. Our analysis of native gels in which the different length templates were hybridized to the RNAs revealed that shorter templates were inefficient at hybridizing to the RNA (12 and 17 nucleotides for $M1^{B}$ RNA) (**Figure S4**). While we do not suggest a specific length of DNA template to bind to the RNA of interest, we recommend optimizations to identify the optimal length based on the RNA of interest.

Supplementary material

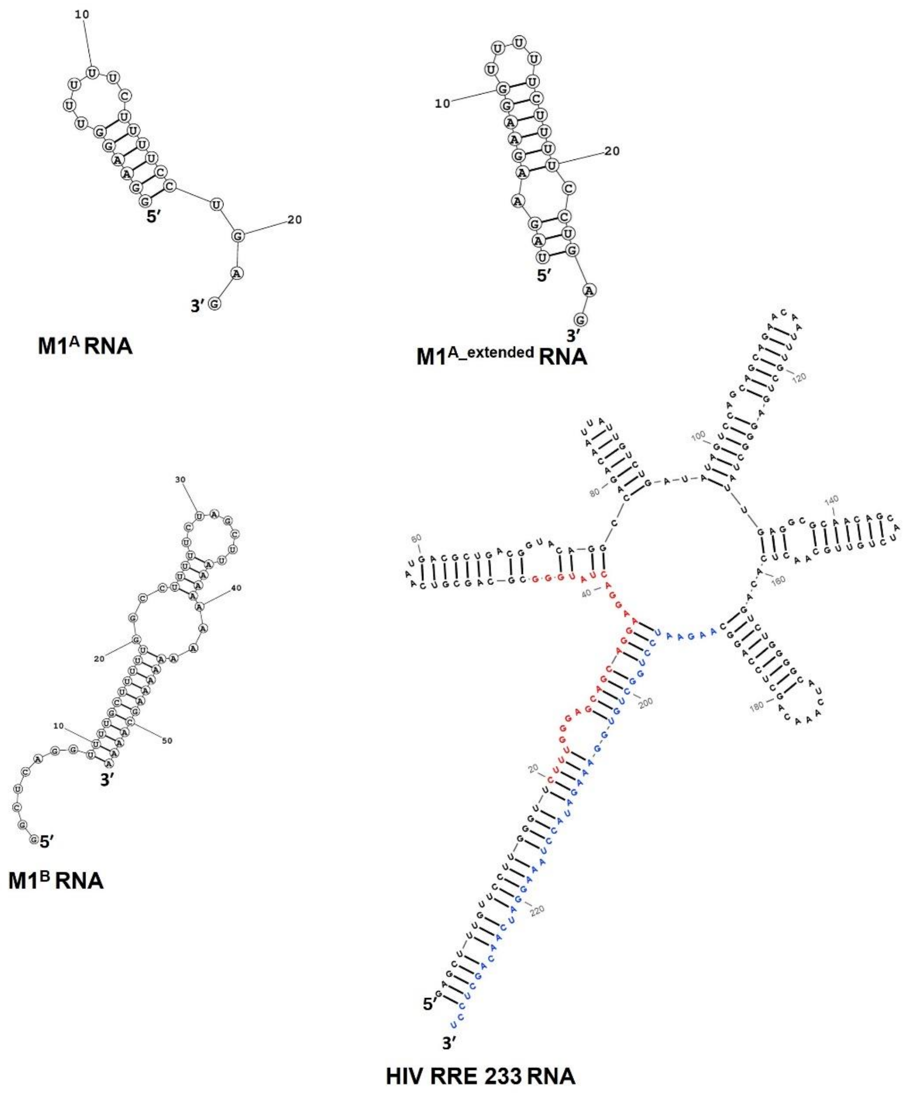

Fig. S1: Predicted secondary structures of RNAs used in this study. *$M1^{A\_shortened}$ (16 nucleotides) RNA does not have a predicted structure. $M1^{A}$ (22 nucleotides), $M1^{A\_extended}$ (26 nucleotides), and $M1^{B}$ (54 nucleotides) have simple, hairpin-like structures. The 233-nucleotide HIV RRE RNA has a more complex structure of 6 stems around a central junction. There is a long stem towards the terminal end of the HIV RRE RNA. The nucleotides complementary to the DNA template are color-coded in blue, and the DNA capture oligonucleotides are color-coded in red, for the HIV RRE RNA. All structure predictions were done using the RNAstructure software.*

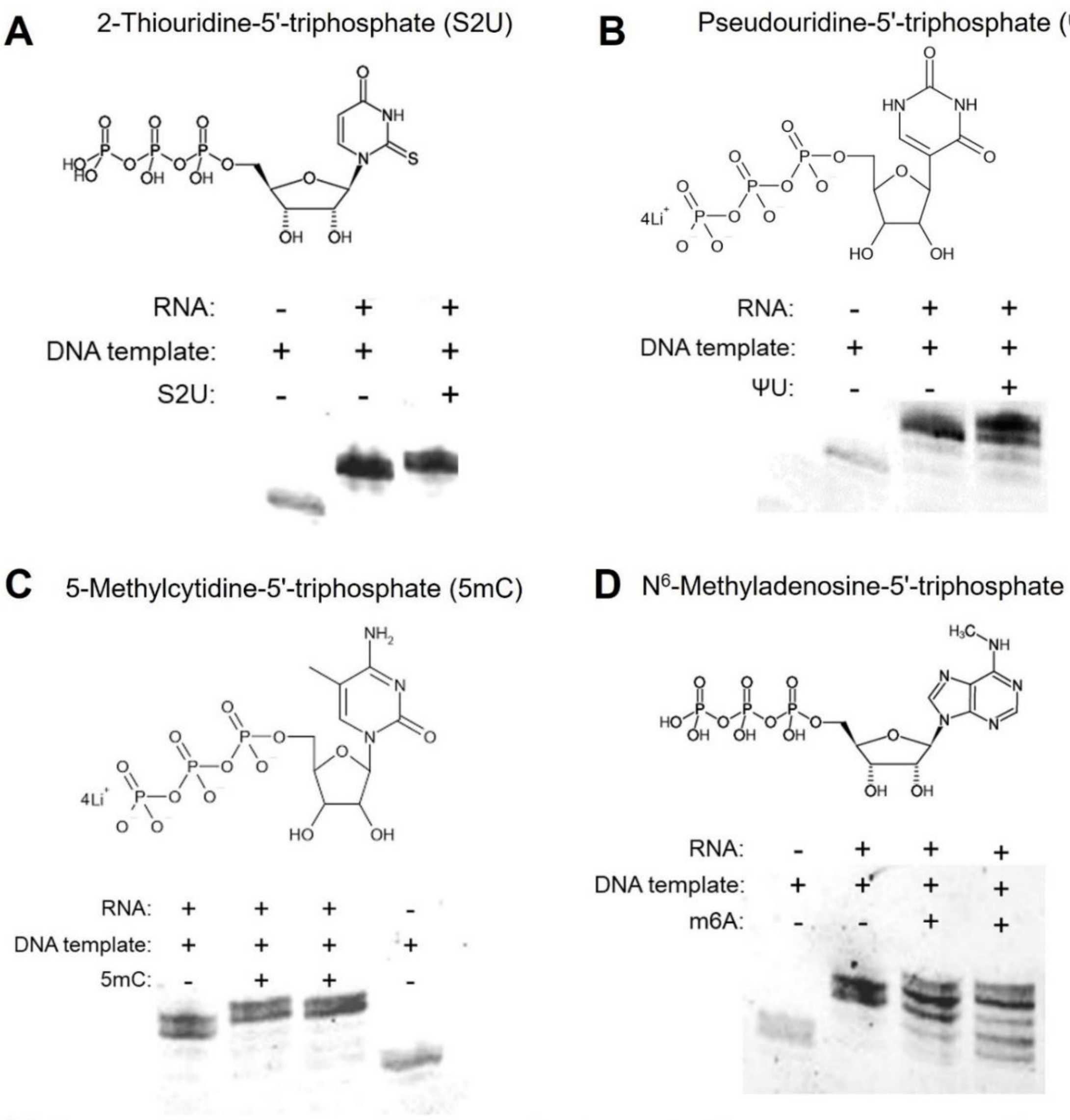

E

	2h-1000uM [Nu]
AA-dUTP	>99% incorporation
2-Thio-UTP	>99% incorporation
Pseudo-UTP	<99% incorporation
N6-methyl-ATP	Degradation with minimal incorporation
5-methyl-CTP	>99% incorporation

Fig. S2: Preliminary data showing the templated addition of modified nucleotides *2-thiouridine, pseudouridine, 5-methylcytidine and N^6-methyladenosine (**A-D** respectively) by Klenow DNA polymerase to the 3' end of RNA. The n+1 addition reaction contained 1 mM of the respective nucleotide and was incubated for 2-16 hours. It is important to note that the preliminary data presented in this figure suggests degradation of the RNA under the tested conditions (**A**, **B**, and **D**) and aberrant gel running conditions (**B** and **D**). However, despite these limitations, the figure suggests promising results for the Klenow n+1 addition reaction with modified nucleotides. Further optimization is expected to improve the incorporation efficiency of these and other modified nucleotides.* ***E.*** *Table summarizing the addition of different modified nucleotides in an n+1 addition reaction employing Klenow with a 2-hour incubation.*

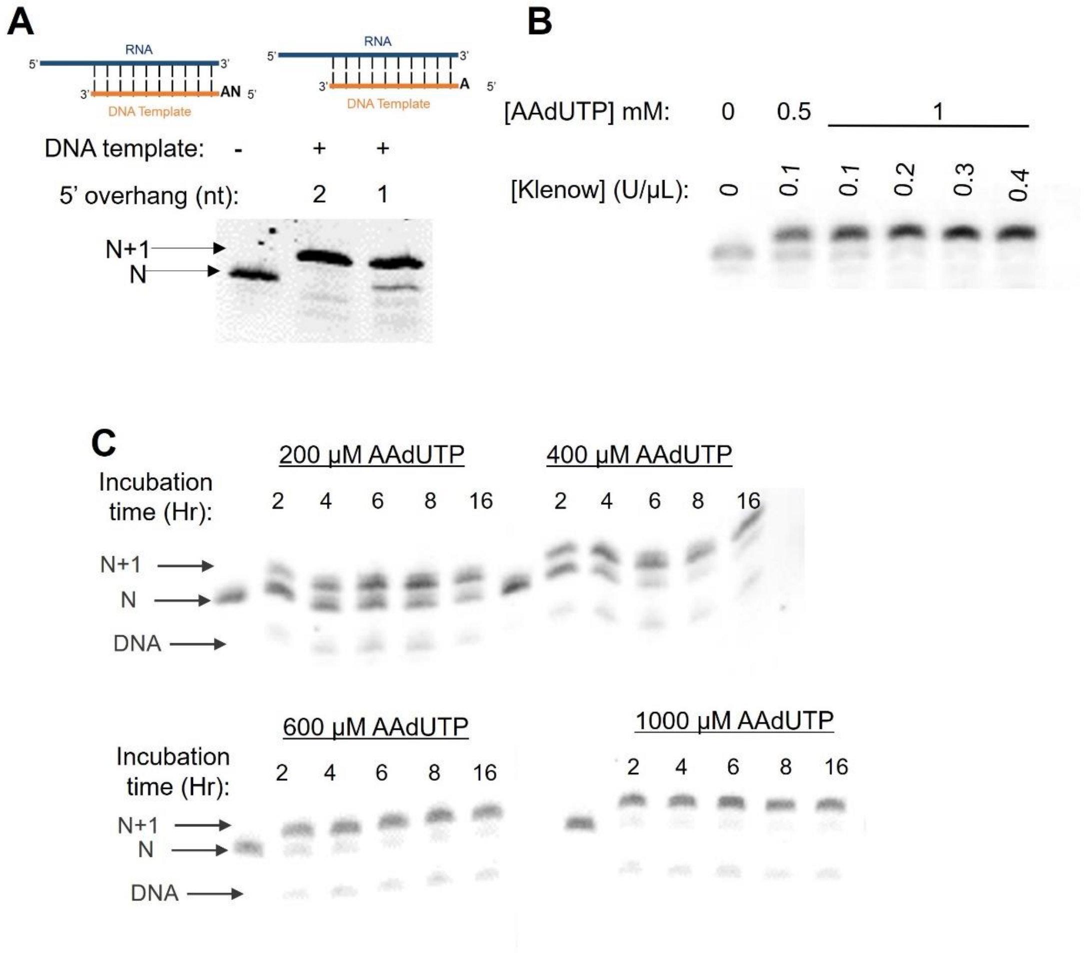

***Fig. S3: Summary of the optimizations for the 3' addition of AAdUTP to RNA**. All experiments in this series were carried out using the model RNA M1A (22 nt) at 2.67 μM in a final volume of 25 μL with 1-hour of incubation at 37°C unless otherwise stated. The n+1 reactions analyzed on the 20% denaturing analytical gels. **A**. Comparison of the n+1 addition reaction efficiency when the 5' DNA template overhang is either 1-nucleotide or 2-nucleotides. **B**. The n+1 addition reaction at increasing concentrations of enzyme. **C**. Comparing the efficiency of the n+1 addition reaction with increasing incubation time and AAdUTP concentration. The 200μM and 400μM samples appears slightly twisted, which may be due to a minor issue during gel electrophoresis. However, the interpretation of the trends in the migration is not impacted because there is a significant shift in the bands upon addition of the AAdUTP and increased incubation time.*

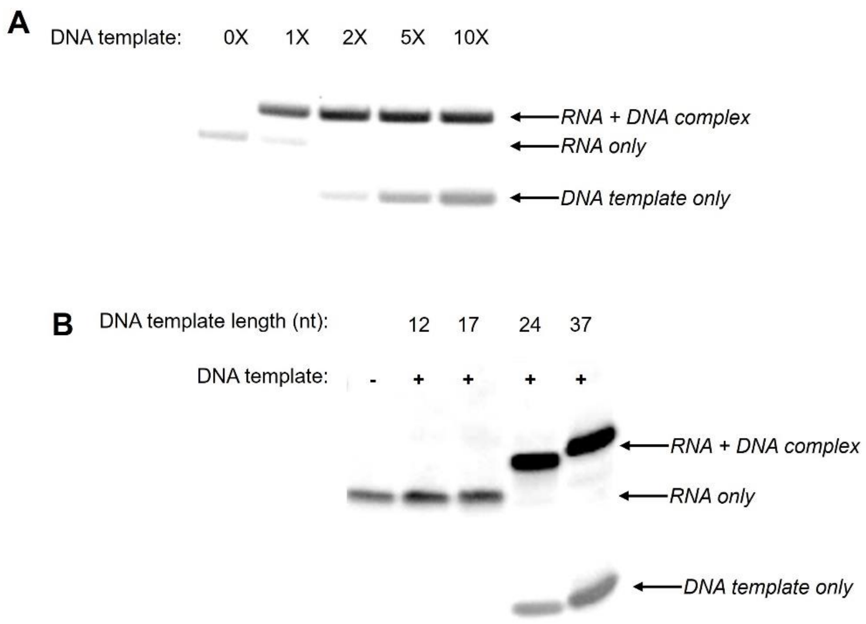

Fig. S4: Summary of optimizations of DNA template concentration and length in M1^B RNA. A. *A native gel shift analysis was used to test the efficient hybridization of the 37 nucleotide DNA template with the M1^B RNA. The DNA template concentration was increased from a 1-fold to a 10-fold excess of the RNA. Upon increasing the concentration of the DNA template, an RNA-DNA complex was formed.* ***B.*** *A native gel shift assay to test how the length of the DNA template at a 2-fold concentration affected the annealing of the template to the M1^B RNA at room temperature incubation. For the shorter DNA templates (12 and 17 nucleotides), the RNA band was not shifted, suggesting inefficient hybridization under our tested incubation temperature. However, for the longer DNA templates (24 and 37 nucleotides), the RNA band was completely shifted, indicating hybridization of the DNA template with the RNA.*
